# Synergistic and Antagonistic Effects of Aerosol Components on Its Oxidative Potential as Predictor of Particle Toxicity

**DOI:** 10.3390/toxics10040196

**Published:** 2022-04-16

**Authors:** Maria Chiara Pietrogrande, Luisa Romanato, Mara Russo

**Affiliations:** Department of Chemical, Pharmaceutical and Agricultural Sciences, University of Ferrara, 44121 Ferrara, Italy; luisa.romanato@edu.unife.it (L.R.); mara.russo@unife.it (M.R.)

**Keywords:** atmospheric aerosol, oxidative potential, interactions between components, synergistic and antagonistic effects, urban air quality

## Abstract

Quantifying the component-specific contribution to the oxidative potential (OP) of ambient particle matter (PM) is the key information to properly representing its acute health hazards. In this study, we investigated the interactions between the major contributors to OP, i.e., transition metals and quinones, to highlight the relative effects of these species to the total OP. Several synergistic and antagonistic interactions were found that significantly change the redox properties of their binary mixtures, increasing or decreasing the values computed by a simple additive model. Such results from the standard solutions were confirmed by extending the study to atmospheric PM_2.5_ samples collected in winter in the Lombardia region, a hot spot for air pollution in northern Italy. This work highlights that a solid estimation of oxidative properties of ambient PM requires an interaction-based approach accounting for the interaction effects between metals and quinones.

## 1. Introduction

Recently, mounting evidence has been accumulating indicating that particulate matter (PM) triggers adverse health effects through the formation of reactive oxygen species (ROS) or the oxidation of biomolecules [1,2,3,4,5]. 

Inhaled PM can carry PM-bound reactive oxygen species (ROS) on the surface of the lung, where they react with lung-lining fluid antioxidants, or directly introduce redox-active PM species into the human body, so that they can react with biological reductants and generate ROS in vivo [6,7,8,9]. The presence and production of ROS overwhelming the antioxidant’s defenses can cause oxidative stress, leading to cell and tissue damage and induction of inflammatory response [3,5,8,10,11]. Thus, the parameter oxidative potential (OP) has been proposed as a measure of the intrinsic ability of ambient air PM to oxidize target molecules and generate ROS, as an improved exposure metric to be used in epidemiological studies, compared to conventional particle metrics such as mass, surface area, or number. Oxidative properties have gained increasing attention as the first step in the elucidation of the subsequent downstream processes generating adverse health effects, mainly multiple cardiorespiratory outcomes, as reviewed by some authors [1,5,6,10,11,12].

Among multiple cellular and cell-free/abiotic assays developed to quantify OP, the acellular methods are the most frequently used techniques on a laboratory bench scale, because they are low-cost and easy to operate, provide a fast output under an easily controlled environment, and have high repeatability [1,13,14,15,16,17,18,19,20,21]. In general, these assays involve the incubation of PM extracts with chemical reagents and probes, and the response is recorded over time after incubation, as the depletion of reductant surrogate, such as the dithiothreitol (DTT) [22,23,24,25,26], or of a single specific antioxidant, such as ascorbic acid (AA) [27,28,29], or of a composite solution, as synthetic respiratory tract-lining fluid (RTLF) [8,20,21,30,31,32]. Acellular OP has been suggested as a promising screening method to predict the biological reactivity of particles, describing their toxic potential with a unique parameter, which integrates emission sources, size distribution, and/or chemical composition [7,12,30,33,34].

Several studies conducted univariate and multivariate regression analysis to identify the main PM chemical components that drive oxidative properties and demonstrated that the most active species are redox-active transition metals (e.g., Fe, Cu, Cr), redox cycling quinoids, and some major PM constituents (e.g., humic-like substances) [13,14,20,21,35,36,37,38,39]. Even though the coexistence of metals and organics is widely accepted, the relative contribution of various chemical components in the overall OP is still a matter of debate, since PM is a highly complex and variable mixture of inorganic and organic compounds, widely varying in size, shape, and surface area, as well as chemical composition. Most studies have tried to reconstruct the total oxidative properties of PM using a simple additive approach, based on the independent contribution of the individual components, but evidence has been reported that this approach is flawed, as PM components have been found to interact and generate both synergistic and antagonistic effects, that alter the overall redox properties of their mixtures [8,40,41,42,43,44]. Therefore, ignoring these interactions could lead to erroneous estimates of the total OP of ambient PM and of the contribution of various PM components in ROS generation. Similarly, synergistic interactions or cocktail effects of chemicals in mixtures have been reported for biological and toxic activity, as some chemicals can enhance the effect of other compounds, so that they jointly exert a larger effect than predicted. For example, synergy occurred for pesticides and antibiotics, which can interfere with metabolic degradation of other xenobiotic enhancing environmental risk [2,45]. Recently, a limited study was performed to investigate the effects of PM component interaction on OP activity, each of them concerning a single OP assay. For example, the DTT response was found to be enhanced by synergic interactions of Fe with Cu and with quinones [41,42,43], while the AA assay activity was suppressed in binary mixtures of quinones with metals [44]. 

In this study, we investigated the interactions among four target redox-active components of ambient PM in order to highlight the way how these species whether suppress or enhance the oxidative properties of their mixture, measured using both the DTT and AA acellular assays. Two metals and two quinones were selected to represent the redox active species which have been found to mostly contribute to PM oxidative potential [9,36,37,38,39,46,47].

First, we analyzed the interaction effects of simple binary mixtures of laboratory standards, using different concentrations to simulate abundance ratio present in ambient air PM samples. Then, we applied the study to atmospheric PM_2.5_ samples, retaining the compositional complexity of real-world PM particles.

## 2. Materials and Methods

### 2.1. Chemicals and Materials

Sodium phosphate (NaH_2_PO_4_, CS Chemicals, Gujarat, India) and disodium hydrogen phosphate (Na_2_HPO_4_) were purchased from Fisher Scientific (Rodano (Milan), Italy). The 0.1 M phosphate buffer was prepared at pH 7.4 using ultrapure water with resistivity = 18.2 MΩ/cm (Milli-Q® IQ 7000 water purification system, Merck KGaA, Darmstadt, Germany). Then, it was treated with Chelex® 100 sodium form resin (Bio-Rad, Segrate (Milan), Italy) to remove any metal contamination. 

Solutions of DTT and DTNB (5,5’-Dithiobis(2-nitrobenzoic acid) (Sigma Aldrich) were prepared at a concentration of 0.01 M in the 0.1 M phosphate buffer (Na_2_HPO_4_ and NaH_2_PO_4_) at pH 7.4. Solutions of L-ascorSbic acid sodium salt (Sigma Aldrich s.r.l., Milan, Italy) were prepared in ultrapure water at the same concentration 0.01 M. Aqueous solutions of the reagents are unstable at room temperature and sensible to light, and thus they were preserved in amber glass vials in the dark at −20 °C.

Pure standards of the investigated compounds—copper (II), iron (III), 9,10-phenantrenequinone (PNQ) and 1,2-naphthoquinone (NPQ)—were purchased from Merck Life Science, Milan, Italy. Individual standard stock solutions were prepared for each analyte by weighting at 0.01 M concentration, using acetonitrile (for quinones) or MilliQ water (for metals) as solvent. The solutions were stored in a freezer at −20 °C in plastic (metals) and amber glass vials (quinones) and discarded after a month.

### 2.2. Sampling and Characterization of Ambient PM_2.5_

The investigated PM_2.5_ were collected by the ARPA Lombardia (Environmental Monitoring Sector, Milan, Italy) in February 2020 at the urban traffic station Milano Marche, located in the northeastern part of the external ring road (Lat 45°29′46.76″N, Long 9°11′27.43″E), characterized by heavy traffic. Daily PM_2.5_ samples were collected on Teflon (Pall) filters (47 mm diameter) and submitted to gravimetric analysis to measure the aerosol total mass concentration, following the123 EN12341:2014 reference method [48,49]. Elements, including metals Fe and Cu, were determined on Teflon membranes by energy dispersive X-ray fluorescence (ED-XRF) using an Epsilon 4 spectrometer by Malvern Panalytical (Malvern, UK). Proper measuring conditions were chosen to optimize the sensitivity for the two elements. By comparing the filter yield with a set of certified standards good accuracy within 5% was obtained [50].

### 2.3. Assessment of the PM Oxidative Potential 

OP was quantified using the DTT and AA assays, following the procedure described elsewhere [14,28,51]. Spectrophotometric measurements were performed with a UV-Vis spectrophotometer (Jasco V-730, JASCO EUROPE s.r.l., Lecco, Italy) with a 1 cm path length optical cell. Polystyrene and quartz cuvette were used for DTT and AA assays, respectively. 

In the DTT assay, 30 µL of the 0.01 M DTT solution were added to the sample (i.e., time zero) and the rate of DTT depletion (OP^DTT^) was measured as follows. At defined times, a 0.50 mL aliquot of the reaction mixture was removed, and the reaction stopped with trichloroacetic acid (0.50 mL of 10 %). Then, the remaining DTT was reacted with DTNB (5,5′-Dithiobis (2-nitrobenzoic acid)) to generate DTT-disulphide and 2-nitro-5-thiobenzoic acid (TNB): 50 μL of the DTNB solution (0.01 M concentration in phosphate buffer at pH 7.4) was added to each aliquot and well mixed. After two minutes to allow the complete reaction, pH was increased to pH 8.9 by adding 2.0 mL of Tris-HCl buffer (0.40 M at pH 8.9 with 0.02 M of EDTA) to form the mercaptide ion (TNB^2-^), which was determined by light absorption at 412 nm (molar extinction coefficient ε = 14,150 M^−1^ cm^−1^).

In the AA assay, 30 µL of the 0.01 M AA solution were added to the sample (i.e., time zero). Then, the rate of AA depletion (OP^AA^) was followed directly in the spectrophotometric cuvette by measuring the absorption of the ascorbate ion at 265 nm (ε = 14,500 M^−1^ cm^−1^ at pH 7.4) at defined time intervals.

The rate of DTT or AA depletion (nmol min^−1^) was determined by linearly fitting the experimental points of the reagents concentration versus time.

#### 2.3.1. Analysis of Standard Solutions

Measurements were performed on 3 mL of laboratory solutions of individual standards or binary mixtures obtained by diluting the stock solutions into 3 mL of phosphate buffer and incubated at 37 °C. The response of blank solutions was determined and subtracted from the data of samples. All tests were performed in triplicate and the results are reported as mean ± standard deviation values.

#### 2.3.2. Analysis of PM_2.5_ Samples

A quarter of the PM filter was extracted for 15 min in an ultrasonic bath using 10 mL of the phosphate buffer 0.1 M at pH 7.4. The extract was then filtered on a regenerate cellulose syringe filter (13 mm, 0.22 mm, Kinesis) to remove the suspended solid particles. Then, 3 mL of the solution were submitted to each OP assay. The response of blank filters was determined and subtracted from the data of samples. For each PM_2.5_ sample, the obtained OP responses are expressed as volume-normalized OP_V_ ^DTT^ and OP_V_^AA^ with reference to the air collected volume (23 m^3^), as a suitable parameter for PM population exposure and inhalation assessment studies.

### 2.4. Statistical Analyses

For each binary mixture of different substances (A and B), the interaction factors (IFs) were calculated to represent the synergistic/antagonistic interactions between the components [41,42]. IF is defined as the ratio of the OP activity (either DTT or AA consumption) experimentally measured on the mixture, *OP_exp_*, and the value computed by summation of individual activities of the respective substances, *OP_calc_*, obtained by neglecting any interactions among the mixture components, according to Equation (1):

(1) $${\mathrm{IF}}_{\mathrm{AB}}=\frac{OP_{exp}}{OP_{calc}}$$

Interactions between the components are identified by IF values significantly different from 1, (*p* < 0.05; independent samples *t*-test), which corresponds to the linear additivity. In particular, synergistic and antagonistic effects amplifying and diminishing the mixture *OP_exp_* are defined for IFs greater or lower than 1, respectively. All the interaction experiments were conducted in triplicate except those involving the ambient PM samples, which were analyzed only once.

## 3. Results

In this study, we chose two acellular DTT and AA assays, since they are the most commonly used and have shown significant associations with adverse health outcomes in some studies [12,13,14,15,16,17,18,19,23,24,25,26,52]. Since they are based on different target antioxidants, they display different sensitivity for each single species, although they are reactive towards the same redox-active species [17,19,28,52]. This sensitivity can be quantified by the slope of linear relationship between the OP^DTT^ and OP^AA^ response and the species concentration, which has been generally found to be within the concentration range of environmental relevance, i.e., from 0.25 to 2 mM (mM concentration indicates the number of mmole per liter). The only exception has been reported for OP^DTT^ response for Cu, as linearity has been found to be true only in a limited concentration range up to 1 mM [21,53].

Our previous results [10,28] showed that, of the investigated species, PQN is by far the most reactive species to the DTT assay, being nearly 10 times more reactive than the other species, according to the hierarchy:

PQN > > Cu ~ NPQ > Fe.

Otherwise, Cu and NPQ are the most active species towards the AA response, showing a sensitivity nearly 10 times higher than those of Fe and PQN, following the order:

Cu > NPQ > > Fe ~ PQN.

We first measured the OP^DTT^ and OP^AA^ responses of each target compound at two or three different concentrations, from 0.1 to 3 µM (µM concentration indicates the number of µmole per liter). They were selected depending on the different reactivity of each individual species, in order to guarantee the best accuracy and precision of the data, which were obtained for OP values ranging from 0.5 to 5 nmol min^−1^ [11,17,26,28,53]. In fact, these values allow an accurate linear interpolation of the relationship of concentration vs. time during the kinetic study duration (40 min). Each measure was performed in triplicate and the obtained OP values are reported as mean and standard deviation in Table 1. Under the operative study conditions, the OP^DTT^ and OP^AA^ responses showed a good precision, described by CV% ≤ 10%.

Then, we conducted interaction experiments on binary mixtures of the four target compounds in order to explore the possible interactions between the PM components, that can suppress or enhance the mixture oxidative properties. We measured the OP^DTT^ and OP^AA^ responses of each mixture and compared the results with the data from the individual compounds, to compute the interaction factor (Equation (1)). For each binary mixture we investigated two mole ratios between the two components, as it is likely that the nature and strength of interactions are affected by the concentrations of the mixture components, with a dependence that is not necessarily a linear function of their concentrations. The mixture compositions were selected in order to approximate the proportion of metals and quinones in real-word ambient PM exposure (Table 2).

### 3.1. Effects of Combinations of Metals and Quinones on DTT Assay Responses

**Figure 1:**
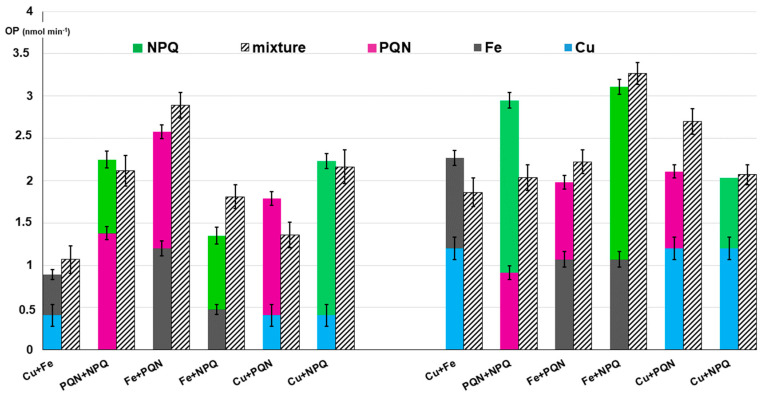
DTT consumption OP (**left** side) and AA consumption (**right** side) activities of Cu, Fe, PQN, NPQ and their binary mixtures at lower molar ratio. OP is expressed as nmol min^−1^. The concentrations of each metal and quinone in the binary mixtures are reported in Table 1, in the first line for each mixture. The colored bars indicate OP of each single compound, while the staked bars show OP experimentally measured on their binary mixtures. Error bars denote standard deviation (1 σ) of triplicate analysis.

The effects of interaction between metals and quinones on DTT activities are reported in Table 2. For the binary mixtures at lower concentration ratios, the OP responses are illustrated in detail in Figure 1 (left side), where colored bars indicate the OP of each single compound, while the staked bars show the OP experimentally measured on their binary mixtures. Based on the calculated IF values, we can observe that Cu and Fe synergistically interact to accelerate DTT oxidation, with the effect increasing with the concentration of Cu in the mixture, from an IF value of 1.20 ± 0.26 to 1.88 ± 0.68 when the Cu:Fe ratio is increased from 0.17 to 1. This synergistic interaction between metals is in agreement with previous experiments by other authors showing an increase in DTT consumption with the addition of Cu at various concentrations of Fe [21,26,40,41,42]. Such an effect was explained on the basis of the mechanism proposed by Kachur, involving the formation of a Cu^2+^-DTT complex, which catalyzes the oxidation of free DTT via the formation of an oxygen-containing intermediate [54]. 

The combination of the two quinones showed an additive effect on DTT oxidation, as the measured IF values were very close to 1. This suggests that the oxidation pathways of two quinones do not significantly interact, although they display different activities, which is in agreement with previous studies [21,26,40,41,42]. 

The mixtures of Fe with PQN and NPQ showed synergic effects, with a general trend of increasing absolute IF values with the Fe:quinone concentration ratio. Of the two quinones, a slightly stronger synergism could be observed for NPQ (1.34 < IF < 1.66) than for PQN (1.12 < IF < 1.32). Similar results have been obtained by other authors, who hypothesized that the synergistic effect of Fe arises from the Fenton reaction, in which H_2_O_2_ generated by quinone-catalyzed DTT oxidation is more efficiently converted to·OH by Fe (II) [26,41,42,44].

On the contrary, the interactions of Cu with quinones were mostly antagonistic in DTT consumption, with a clear interaction for PQN (0.66 < IF < 0.76), while a weak effect close to additivity for NPQ (0.89 < IF < 0.97) was also shown. Our results are consistent with previous studies, which suggested that such OP reduction upon mixing Cu with quinones may be ascribed to metal–organic binding interactions, since Cu can form complexes with quinones, as supported by evidence from proton nuclear magnetic resonance (^1^H NMR) spectroscopy [54,55]. Thus, although the mechanism of interaction between Cu and quinones is not fully understood, it seems that the varying strength of the antagonistic effect depends on the varying ability of Cu to bind with quinones and the variation in molar ratio of Cu to quinone in the mixture [41,43,54].

### 3.2. Effects of Combinations of Metals and Quinones on AA Assay Responses

The interactions between metals and quinones and the AA depletion rate were investigated in detail by measuring the OP^AA^ responses of each individual solution and their binary mixtures at two concentration ratios (IF values in Table 2, OP^AA^ responses of the binary mixtures at lower concentration ratios in the right side of Figure 1). 

The measured OP^AA^ data of the mixtures containing Cu and Fe showed a clear antagonistic interaction between the two metals, with IF values decreasing from 0.82 ± 0.04 to 0.62 ± 0.04 with increasing the Cu:Fe concentration ratio. Similar behavior has been observed in previous papers, in which Cu ions were found to inhibit the ·OH radical generation from the iron-driven Fenton reaction [20,21,27,31,44,56,57,58]. Additionally, the combinations between the two quinones showed antagonistic effects on the AA consumption, as the measured IF values ranged from 0.52 ± 0.07 to 0.69 ± 0.09. To date, no mechanism has been suggested to explain these effects, since the redox interaction between quinones and ascorbate has been poorly investigated.

The interaction experiments measuring the AA consumption of mixtures of metals and quinones showed nearly additive effects for NPQ with both metals, with IF values close to 1, while weak synergic interaction for PQN was observed (Figure 1 and Table 2). In particular, the interactions of PQN with Fe were quantified by IF values ranging from 1.12 to 1.24, while those with Cu showed IF values close to 1.3. These results are consistent with some previous studies, which revealed that traces of metallic ions, especially iron, enhance generation of ROS, in particular, they play an important role in oxidizing ascorbate to ascorbyl radical, as the key step in the redox process with which quinones could begin their redox cycling [27,31,44,55,57,58,59].

### 3.3. Application to Real Ambient PM

We extended the study to eight ambient PM_2.5_ filters collected in the city of Milan, in order to provide insight into the interaction effects among the components of real-world aerosols. On each PM_2.5_ extract, the OP values were measured with both assays; the obtained responses ranged from 0.33 to 1.50 nmol min^−1^ for OP^DTT^ and from 1.01 to 4.83 nmol min^−1^ for OP^AA^ (Table 3). By expressing these results as volume-normalized OP_V_, normalized to collected air volume, they correspond to 0.19–0.87 nmol min^−1^ m^−1^ for OP_V_^DTT^ and to 0.67–2.80 nmol min^−1^ m^−1^ for OP_V_^AA^ under the experimental conditions in this study. Such oxidative properties are consistent with the data previously reported for similar samples in Milan and Northern Italy [14,28,51,60,61,62], as well as in other urban locations strongly influenced by anthropogenic air pollution [9,11,18,23,33,46,63,64].

Most studies, for different geographic locations covering a wide range of PM_2.5_ concentration levels, show that aerosol OP is mainly driven by contribution of metals related to vehicle traffic emissions in the urban atmosphere (70–80%), while organic components (mainly secondary organic compounds, SOA) contribute only ~20% [21,23,36,64]. Among the wide variety of transition metals present in PM, Fe is typically the most abundant, followed by Cu, which is typically 4–15% of Fe concentration [8,13,14,15,16,17,18,19,23,29,33,37,46,63,64]. Therefore, of the different parameters quantified for sample chemical characterization, the concentrations of copper and iron were investigated in detail in this study (Table 3). The data clearly show that, of the two redox active metals, Fe is by far the more abundant in the investigated PMs, with concentration levels 25–30 times higher than those of Cu. The dominating contribution of these metals to the PM oxidation properties was confirmed by the significant correlation of their concentration with OP^DTT^ and OP^AA^ responses, as proven by Pearson correlation coefficients (significant at *p* < 0.005). Their dependence also motivated the significantly higher OP^AA^ responses from AA assay (mean value 3.76 ± 1.37 nmol min^−1^) compared with those from DTT (mean value 0.78 ± 0.42 nmol min^−1^), as the first assay has been found more sensitive to redox-active metals, in particular to Cu (e.g., [14,17,19,27,28,38]). 

**Figure 2:**
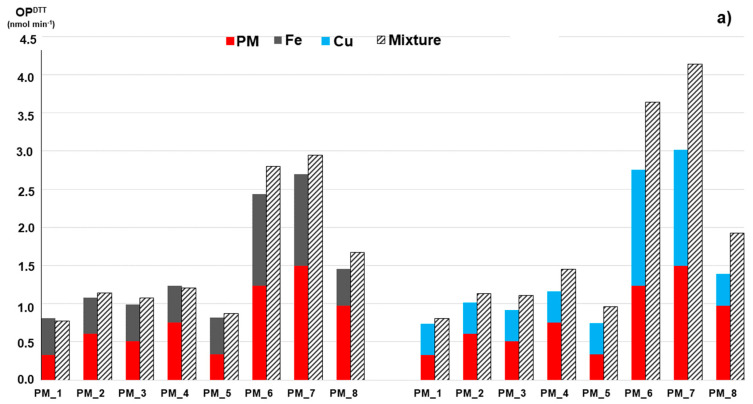

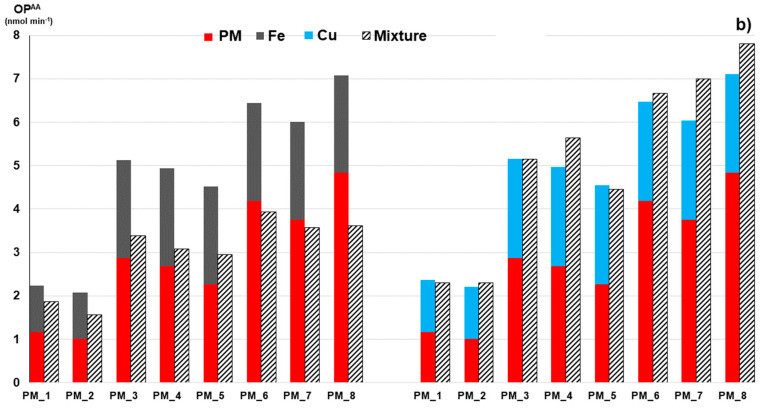
DTT consumption OP^DTT^ (**a**) and AA consumption OP^AA^ (**b**) activities of the real-world PM_2.5_ samples. The bottom red bars indicate OP of each individual PM extract, the top bars are OP of the standard solutions of Fe and Cu added to the extract (grey: Fe, light blue: Cu), while the staked bars show OP experimentally measured on their combinations. OP is expressed as nmol min^−1^. The concentrations of the added Fe and Cu solutions are reported in Table 4.

Interaction experiments were performed by adding known amount of the target metals and quinones to different fractions of the buffer extract of each original PM_2.5_ sample. The added concentrations were chosen to obtain OP responses close to those measured in the original PM samples, in order to simulate real-world samples more closely, and also to guarantee the highest measure accuracy. The IF values were computed by comparing the OP responses experimentally measured on the spiked samples to the values calculated as summation of OP of the original extract and the standard solution (Table 4). Figure 2 illustrates the measured OP responses from these experiments by adding Fe and Cu solutions (grey and light blue bars, respectively) to each PM_2.5_ extract.

The results obtained from the DTT assay clearly showed that Fe addition to the PM_2.5_ extracts generated a simple additive contribution to OP, with IF values close to 1 (0.96 < IF < 1.15, right side in Figure 2a), while Cu mixing significantly increased the OP^DTT^ responses of the mixture, indicating a synergistic effect (1.09 < IF < 1.39, left side in Figure 2a). This interaction behavior is very similar to that found for the Fe standard solutions (Table 2), suggesting that Fe is the dominant species responsible for DTT oxidation in the studied PM_2.5_ samples. This is also confirmed by the finding that the IF values regularly increase with the Cu concentration in the sample, as previously found for the Cu-Fe mixtures. Addition of quinones to PM_2.5_ samples showed synergic effects to the total OP, with lightly stronger effect for NPQ (1.16 < IF < 1.71) than for PQN (1.09 < IF < 1.37) (Table 4). Additionally, these interactions resemble those of Fe standard solutions, thus confirming that Fe was mainly responsible for the DTT activity. 

Then, we investigated the contribution of additional metals and quinones in altering the AA consumption of real PM_2.5_. By comparing the OP responses of each PM extract after addition of the two metals, we can observe a simple additive contribution for Cu, with IF values close to 1 (0.97 < IF < 1.16), while a clear decreasing of the OP^AA^ responses for Fe, indicating an antagonistic effect (0.51 < IF < 0.84) (Figure 2b). This interaction behavior is consistent with that found for the Cu standard solutions (Table 2), suggesting that Cu could be the main component driving the AA oxidation. Concerning the contribution of adding quinones, the obtained data showed an additive effect for both NPQ and PQN, with IF values close to 1, suggesting that the added quinones do not significantly interact with the components of the complex PM mixture (Table 4), similar to what was found for OP^AA^ of the binary mixtures of standard Cu and Fe and quinones (Table 2). These findings suggest that for the study samples, the AA assay responses were mainly dominated by the Cu concentration. 

## 4. Discussion

The obtained results clearly show that the target metals and quinones interact in oxidation reactions, generating a significant change in the mixtures’ redox properties compared with the values computed by a simple additive model. The only exceptions are the mixtures of the two quinones on the DTT oxidation. Fe and Cu mixtures showed a synergistic increase in DTT consumption, while an antagonistic effect on AA oxidation. Mixtures of quinones showed similar antagonistic effects on AA consumption. The interaction effects were also measured in binary mixtures of metals and quinones, as both classes are simultaneously present in the same particles. The combination of metals with quinones yielded additive to weak synergistic interactions for both OP assays, with the exception of Cu + PQN mixtures, showing an antagonistic effect on the OP^DTT^ responses.

By combining the results from both DTT and AA acellular assays, we obtained complementary information on the contribution of PM components on its overall oxidative properties, since each method captures different redox-active species. In particular, such a complementarity of DTT and AA assays was very helpful in the study of real-world samples. By measuring the oxidative properties of samples spiked with redox-active species, we found that the responses of each assay were specifically dominated by one of the most abundant metals in the non-exhaust traffic emissions, i.e., OP^DTT^ by Fe, and OP^AA^ by Cu. These results can be explained by considering that the importance of each species in driving OP responses depends upon its mass concentration, in combination with its intrinsic OP [8,13,15,26,40,53]. The dominant contribution of Fe to OP^DTT^ may be explained by its high abundance in ambient PMs, despite its low sensitivity towards the DDT assay. This result is in agreement with previous studies, which reported that Fe accounted for the majority of DTT loss from several ambient PM_2.5_ and, consistently, that it may be predictive of OP^DTT^ [23,26,31,64]. Actually, the strong correlation between Fe concentration and DTT oxidation has been also related to the suggestion that Fe may represent a surrogate measure of other not quantified compounds co-emitted with Fe from brake and tire wear with intrinsic redox active properties [15,26,47].

The likely explanation for the driving role of Cu on AA oxidation is its having the highest reactivity, despite its relatively modest mass concentration in the ambient PMs in this study. These results are in agreement with our previous studies in Northern Italy, emphasizing the high potential of copper to cause PM oxidative stress [14,28,51,61,64] and with other results in similar sites in Europe [8,15,17,18,19,63,64].

## 5. Conclusions

The results obtained on laboratory standard solutions as well as on real-world PMs clearly showed that the interactions among transition metals and quinones have to be taken into account for an accurate investigation or prediction of OP of atmospheric PM based on its chemical composition. However, it should be noted that the translation of the synergistic or antagonistic results from our simple model mixtures to ambient PM needs caution, since atmospheric samples consist of a wide variety of coexisting components, which may be redox-active themselves or be involved in metal–ligand complexation, changing availability towards oxidation reactions. A deeper insight into these effects would require further experiments to explore a more extended range of components concentrations, also simulating real PM composition more closely. 

The findings of the study, based on both DTT and AA assays, given the specific sensitivity of each assay to the different redox active species, emphasize the importance of combining the two assays for integrating different information on the effects of PM species on OP measures and in turn assess the results of the health studies that use OP.

The mechanistic approach used in this study can be extended to investigate the possible interactions among PM components effecting the adverse health impact, as quantified by several biological end points. However, extrapolation of the results of these interaction experiments to the biological effects is inherently difficult, mainly due the incomplete knowledge of the exact mechanisms through which PM components trigger their biological effects in exposed cells or organisms, and the difficulty of simulating biological conditions in real life using acellular assays. Further studies adapted to different toxicity probes are in progress to investigate synergistic and antagonistic interactions among the PM components as explanatory variables of the total PM biological activity.

## Figures and Tables

**Table 1:** Values of DTT and AA consumption (nmol min^−1^) measured for each individual standard solution at the different concentrations (µM represents the number of µmole L^−1^) used to prepare the binary mixtures.

Standard	OP^DTT^ (nmol min^−1^)	OP^AA^ (nmol min^−1^)
Fe 1 µM	0.48 ± 0.06	1.07 ± 0.09
Fe 2 µM	1.20 ± 0.09	2.25 ± 0.08
Fe 3 µM	1.70 ± 0.11	-
Cu 0.05 µM	-	1.20 ± 0.13
Cu 0.10 µM	-	2.28 ± 0.11
Cu 0.17 µM	0.41 ± 0.13	5.31 ± 0.15
Cu 0.5 µM	1.52 ± 0.06	-
Cu 1 µM	3.03 ± 0.07	-
PQN 0.17 µM	1.38 ± 0.08	-
PQN 0.25 µM	2.51 ± 0.07	-
PQN 0.50 µM	6.26 ± 0.09	0.15 ± 0.10
PQN 1 µM	-	0.91 ± 0.08
NPQ 0.10 µM	-	0.83 ± 0.08
NPQ 0.17 µM	-	2.04 ± 0.10
NPQ 0.25 µM	0.87 ± 0.10	3.20 ± 0.09
NPQ 0.5 µM	1.82 ± 0.09	5.20 ± 0.07
NPQ 1 µM	4.14 ± 0.09	

**Table 2:** Interaction factors (IFs) for DTT and AA consumption in the binary mixtures of metals and quinones. For each species pair, two concentration levels were investigated based on the concentration sets of individual standards reported in Table 1.

Components	Composition of Binary Mixtures	Molar Ratio	IF OP^DTT^
Fe-Cu		Cu/Fe	
	Cu 0.17 µM + Fe 1 µM	0.17	1.20 ± 0.26
	Cu 1 µM + Fe 1 µM	1	1.88 ± 0.68
PQN-NPQ		PQN/NPQ	
	NPQ 0.25 µM + PQN 0.17 µM	0.68	0.94 ± 0.05
	NPQ 0.25 µM + PQN 0.25 µM	1	0.98 ± 0.07
Fe-PQN		Fe/PQN	
	Fe 2 µM + PQN 0.17 µM	11.8	1.12 ± 0.06
	Fe 3 µM + PQN 0.17 µM	17.6	1.32 ± 0.08
Fe-NPQ		Fe/NPQ	
	Fe 1 µM + NPQ 0.25 µM	4	1.34 ± 0.20
	Fe 2 µM + NPQ 0.25 µM	8	1.66 ± 0.07
Cu-PQN		Cu/PQN	
	Cu 0.17 µM + PQN 0.17 µM	1	0.76 ± 0.08
	Cu 0.5 µM + 9PQN 0.17 µM	2.9	0.66 ± 0.05
Cu-NPQ µM		Cu/NPQ	
	Cu 0.17 µM + NPQ 0.5 µM	0.34	0.97 ± 0.04
	Cu 0.5 µM + NPQ 0.5 µM	1	0.89 ± 0.12
Components	Composition of binary mixtures	Molar ratio	IF OP^AA^
Fe-Cu		Cu/Fe	
	Cu 0.05 µM + Fe 1 µM	0.05	0.82 ± 0.04
	Cu 0.1 µM + Fe 1 µM	0.1	0.62 ± 0.04
PQN-NPQ		PQN /NPQ	
	PQN 1 µM + NPQ 0.17 µM	5.9	0.69 ± 0.09
	PQN 1 µM + NPQ 0.1 µM	10	0.52 ± 0.07
Fe-PQN		Fe/PQN	
	Fe 1 µM + PQN 1 µM	1	1.12 ± 0.06
	Fe 1 µM + PQN 0.5 µM	2	1.24 ± 0.08
Fe-NPQ		Fe/NPQ	
	NPQ 0.17 µM + Fe 1 µM	5.9	1.05 ± 0.09
	NPQ 0.1 µM + Fe 1 µM	10	1.12 ± 0.18
Cu-PQN		Cu/PQN	
	PQN 1 µM + Cu 0.05 µM	0.05	1.28 ± 0.12
	PQN 1 µM + Cu 0.1 µM	0.1	1.35 ± 0.10
Cu-NPQ		Cu/NPQ	
	Cu 0.05 µM + NPQ 0.1 µM	0.5	1.02 ± 0.12
	Cu 0.1 µM + NPQ 0.1 µM	1	1.14 ± 0.10

**Table 3:** Oxidative potential and chemical composition of real-word PM_2.5_ samples: concentration of two metals (Cu, Fe) and DTT and AA consumption rate.

Sample No.	OP^DTT^ (nmol min^−1^)	OP^AA^ (nmol min^−^^1^)	Cu (ng m^−3^)	Fe (ng m^−3^)
PM_1	0.33	1.16	0.010	0.27
PM_2	0.60	1.01	0.011	0.32
PM_3	0.51	2.88	0.022	0.55
PM_4	0.75	2.69	0.029	0.75
PM_5	0.34	2.26	0.037	0.97
PM_6	1.24	4.19	0.052	1.35
PM_7	1.50	3.76	0.054	1.58
PM_8	0.98	4.83	0.063	1.60

**Table 4:** Interaction factors for DTT and AA consumption in the real-world PM_2.5_ samples after addition of standard solutions of Cu and Fe metals and of PQN and NPQ quinones.

**IF OP^DTT^**				
**Sample No.**	**+ Fe 1 μM**	**+ Cu 0.17 μM**	**+ PQN 0.17 μM**	**+ NPQ 0.25 μM**
PM_1	0.87	1.09	1.09	1.16
PM_2	1.05	1.12	1.10	1.14
PM_3	1.09	1.21	1.14	1.20
PM_4	0.98	1.25	1.18	1.22
PM_5	1.13	1.28	1.21	1.30
PM_6	1.15	1.32	1.29	1.49
PM_7	1.19	1.37	1.33	1.54
PM_8	1.14	1.39	1.37	1.71
mean	1.10	1.25	1.23	1.35
**IF OP^AA^**				
**Sample No.**	**+ Fe 2 μM**	**+ Cu 0.1 μM**	**+ PQN 1 μM**	**+ NPQ 0.1 μM**
PM_1	0.84	0.97	1.07	1.07
PM_2	0.75	1.04	1.14	1.41
PM_3	0.66	1.00	1.09	1.09
PM_4	0.62	1.14	0.82	0.96
PM_5	0.65	0.98	0.68	0.84
PM_6	0.61	1.03	0.82	0.82
PM_7	0.59	1.16	0.78	0.85
PM_8	0.51	1.10	1.04	1.04
mean	0.65	1.05	0.93	1.01

## Data Availability

The data presented in this study are available on request from the corresponding author.

## Abbreviations

AA	ascorbic acid
CV%	% coefficient of variation to quantify precision of a measure
DTNB	5,5′-dithiobis(2-nitrobenzoic acid)
DTT	dithiothreitol
^1^H NMR	proton nuclear magnetic resonance
IF	interaction factor
mM	concentration unit indicating the number of mmole per Liter
µM	concentration unit indicating the number of µmole per Liter
NPQ	1,2-naphthoquinone
OP	oxidative potential
OP^AA^	rate of ascorbic acid depletion
OP_calc_	OP activity computed by summation of individual activities of the mixture components,
OP^DTT^	rate of dithiothreitol depletion
OP_exp_	OP activity experimentally measured on a mixture
OP_V_	volume-normalized OP response referred to air collected volume
OP_V_^AA^	volume-normalized OP^AA^ response
OP_V_^DTT^	volume-normalized OP^DTT^ response
PM	particle matter
PM_2.5_	particles with diameters of 2.5 µm and smaller
PNQ	9,10-phenantrenequinone
ROS	reactive oxygen species
RTLF	respiratory-tract lining fluid
SOA	secondary organic compounds
TNB	2-nitro-5-thiobenzoic acid
TNB^2-^	mercaptide ion
